# Interfacial Synthesis of an Ultrathin Two-Dimensional Polymer Film via [2 + 2] Photocycloaddition

**DOI:** 10.3390/molecules28041930

**Published:** 2023-02-17

**Authors:** Yanqi Ban, Hui Wang, Zixuan Xiao, Lishui Sun, Qingyan Pan, Yingjie Zhao

**Affiliations:** 1 Engineering Research Center of High Performance Polymer and Molding Technology, Ministry of Education, College of Polymer Science and Engineering, Qingdao University of Science and Technology, Qingdao 266042, China; 2 College of Chemical Engineering, Qingdao University of Science and Technology, Qingdao 266042, China

**Keywords:** two-dimensional polymers, covalent organic frameworks, porous materials, interfacial synthesis, [2 + 2] cycloaddition, photochemistry

## Abstract

A carbon–carbon-linked, ultrathin, two-dimensional (2D) polymer film was prepared at the air/water interface through photochemically triggered [2 + 2] cycloaddition. The preorganization of the monomers on the water surface and the subsequent photo-polymerization led to the successful preparation of the ultrathin 2D polymer film. The obtained film is continuous, free standing, and has a large area (over 50 μm^2^). Transmission electron microscopy (TEM) and atomic force microscopy (AFM) give clear evidence of the ultrathin film morphology. Raman spectroscopy and X-ray photoelectron spectroscopy (XPS) indicate successful photo-induced [2 + 2] polymerization.

## 1. Introduction

Two-dimensional (2D) polymers are single- or few-layered and free-standing covalent networks with long-range order in two directions [1,2,3,4,5,6,7,8]. In contrast to other traditional 1D polymers, 2D polymers have drawn lots of attention due to their unique arrangement of the structural units in two directions, ultrathin thickness, uniform pore size, and facilely-tailored functionality [8,9,10,11,12]. More importantly, the endless flexibility in designing monomers can increase the variety of 2D polymers and endow them with peculiar physicochemical properties [13,14]. In recent years, 2D polymers have been developing rapidly, and these can be widely used in chemical sensors, membrane separation, catalysis, energy conversion and storage, etc. [15,16,17,18,19,20,21,22,23,24]. Therefore, the study of various 2D polymers has important research significance and a high application value. However, the preparation of highly ordered and large-sized 2D polymers is still a challenge, especially in the case of mono- or few-layered 2D polymer films [25,26,27,28,29]. The emergence of 2D covalent organic frameworks (COFs) solved the problem of the periodicity in the 2D structures to some extent. The utilization of dynamic covalent chemistry facilitates the self-adjusting process during the polymerization, and thus, leads to the highly ordered 2D structure via solvothermal synthetic methods [30,31]. The 2D COFs are usually obtained as solid powders [32,33]. The poor solubility and processibility of bulk 2D COFs are appreciable disadvantages limiting their practical application to a certain extent, particularly in thin films or membranes. This is a big disadvantage for their broad practical applications [34].

The study of 2D COFs films is still in its infancy [35]. In contrast to the various documents on bulk 2D COF powders, examples of 2D COFs films have been relatively infrequently reported [36]. There are currently two main strategies for the preparation of 2D COFs films: top-down and bottom-up approaches [19]. Inspired by the graphene sheets from the exfoliation of graphite, the top-down approach is one attractive method for the directly exfoliated bulk 2D COFs powders [37]. At present, there are many ways to obtain free-standing films, such as solvent-assisted exfoliation, mechanical delamination, and self-exfoliation [38,39,40]. However, bulk lamellar COFs have compact stacking forms and narrow interlayer spacing due to the strong interlayer interactions, which make their exfoliation challenging and demanding [41,42]. The alternative bottom-up approach thus becomes an important strategy that enables 2D COFs to be deposited on specific substrates or prepared at interfaces with a controllable thickness. In most cases, the bottom-up approach is a technically simple and powerful tool towards achieving potential applications. More and more methods are being presented as time goes on, including solvothermal synthesis, interfacial polymerization, synthesis under continuous flow conditions, and room temperature vapor-assisted conversion [19]. Additionally, under suitable experimental conditions, 2D structures that can be generated by these approaches can assume very large lateral dimensions. They are essentially based on the direct combination/reaction of different building blocks, allowing the formation of two-dimensional structures. Typically, this can be either carried out on the substrate surface or at the liquid/liquid or air/liquid interface [43,44]. In this case, interfacial polymerization has emerged as the most popular method, and it features a well-defined interface of two phases to guide the formation of continuous, mono-, or few-layered ultrathin 2D COFs or 2D polymer films under mild conditions [45,46]. Interface systems are abundant and suitable for the growth of a wide range of materials. Benefiting from the enhanced chemical reactivity at the interface, some reactions could be realized by interfacial synthesis, whereas these reactions are challenging under solvothermal conditions [47].

According to previous reports, several reaction types have been successfully applied to the interfacial synthesis of 2D COFs or 2D polymer films, such as Schiff base condensation, azide-alkyne cycloadditions, Glaser-Hay coupling reactions, [2 + 2] photocycloaddition, and so on [48,49,50,51]. Dichtel’s groups reported the interfacial polymerization of monomers capable of forming imine-linked 2D COF films at liquid/liquid interfaces. The 2D COF films were transferred onto supports, and the resulting films showed the enhanced rejection of Rhodamine [52]. At the interface of the two phases, Nishihara’s groups prepared a 1,2,3-triazole-linked 2D nanosheet for the storage of metal ions, and they also produced crystalline 2D graphdiyne nanosheets [44,49]. The obtained 2D polymer films have exhibited superior potentials in various applications due to their increased specific surface area, more facile charge transport properties, and more exposed active sites, etc. [53,54,55,56]. Additionally, water with low surface roughness could provide a perfect template or substrate for the confined polymerization in a 2D plane, which is widely available. Photocatalytic cycloaddition reactions based on anthracene dimerization were successfully realized by Schlüter’s and King’s groups at the air/water interface [57,58]. A cycloaddition reaction is a powerful tool for the synthesis of cyclic structures in a green way. In the meanwhile, photocatalytic cycloaddition reactions are also becoming popular due to their high synthetic utility under simple operational conditions. O of the most important photochemical reactions in organic photochemistry is [2 + 2] photocycloaddition, which is conveniently achieved through direct excitation or sensitization by UV irradiation. The cyclobutene derivatives could be prepared by [2 + 2] photocycloaddition, which cannot be obtained in some other way [59,60,61,62,63]. Inspired by these, we considered preparing one carbon–carbon-linked 2D polymer film by photocatalytic cycloaddition reactions.

Here, we prepared a large-sized, ultrathin, and free-standing 2D polymer (**P1**) film at the air/water interface via [2 + 2] photocycloaddition. The monomer (**M1**) was designed as a star-type molecule with *C3* symmetry, which would achieve head-to-tail self-assembly through D-A and π-π interactions between arene and fluoroarene at the water interface. Weak supramolecular interactions as important forces could effectively control the molecular arrangement, which in turn forms an ordered 2D structure. Water was used as the liquid template due to its extremely low surface roughness, and it could confine polymerization at the same time [64]. The subsequent light-intermediated intermolecular [2 + 2] photocycloadditions connected the monomers together in a 2D plane through covalent bonds, and thus, led to the successful construction of 2D polymer films. Raman spectroscopy and X-ray photoelectron spectroscopy (XPS) proved the successful bond formation of the as-prepared 2D polymer film. Transmission electron microscopy (TEM) and atomic force microscopy (AFM) give clear evidence of the ultrathin film morphology.

## 2. Results and Discussion

**Figure 1:**
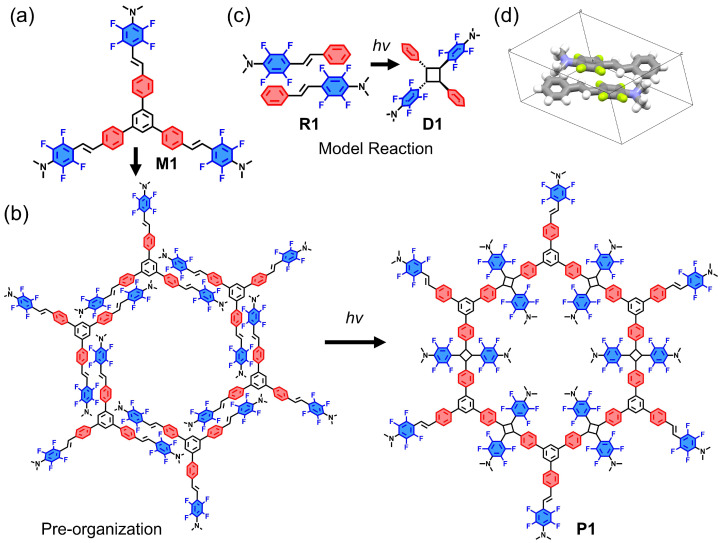
(**a**) The structure of monomer **M1**. (**b**) The self-assembly process of **M1** at the air/water interface and the polymerization under laser irradiation. (**c**) The structure of **R1** and the corresponding dimerization of **R1** to **D1**. (**d**) The single crystal structure of **R1**.

The star-type monomer **M1** (Figure 1a) was synthesized by a heck reaction between 1,3,5-tris(4-iodophenyl)benzene as the core and 2,3,5,6-tetrafluoro-*N*,*N*-dimethyl-4-vinylaniline as the arms. For the synthesis details, see the Supporting Information (Scheme S1). The solubility and reactivity of the monomer are improved by the introduction of *N*,*N*-dimethyl groups. Fluorinated aromatic rings in **M1** contributed to the arene-fluoroarene interactions in the self-assembly process, which in turn promoted [2 + 2] photocycloaddition (Figure 1b). To verify the feasibility, the model compound (**R1**) was synthesized according to the previously reported work (Figure 1c). A single crystal of **R1** was easily acquired through the slow evaporation of its CH_2_Cl_2_ solution. The X-ray single crystal diffraction analysis demonstrates that **R1** adopts a head-to-tail stacking mode in the single crystal state (Figure 1d). The further irradiation of the **R1** solution under a 450 nm LED lamp leads to the dimer **D1** through successful [2 + 2] photodimerization (Figure 1c). According to the suitable self-assembly mode (head-to-tail) of the reference **R1** and the successful realization of the reference dimer **D1** through photo-dimerization, the reasonable preorganization and the corresponding polymerization of **M1** should be feasible.

The 2D polymer film **P1** was then successfully prepared at the air/water interface. The star-type **M1** solution in dichloromethane (250 μL, 1 × 10^−5^ M) was spread uniformly on the surface of the water in a Langmuir–Blodgett (LB) trough. With the volatilization of the dichloromethane, the self-assembly process of **M1** at the air/water interface was investigated by Brewster Angle Microscopy (BAM) [65]. Initially, no surface pressure was observed on the interface due to the loose distribution of **M1**. With the slow shift of barriers, the value of the surface pressure increased gradually as the **M1** molecules assumed a compact arrangement (Figure S1). In the end, uniform and dense films were successfully observed by BAM at the air/water interface (Figure S2). The turning point of the surface pressure appeared at around 41 mN·m^−1^, which indicated that the self-assembly process of monomers at the interface was completed (Figure S2). Furthermore, the value of the measurement of mean molecular area (MMA) value was calculated to be 610 Å^2^/molecule. Subsequently, the pre-assembled monomers were polymerized under irradiation using a 450 nm LED lamp (36 W) for 2 h. Immediately afterward, the obtained films were simply transferred to SiO_2_/Si wafer and copper TEM grids for further characterization.

**Figure 2:**
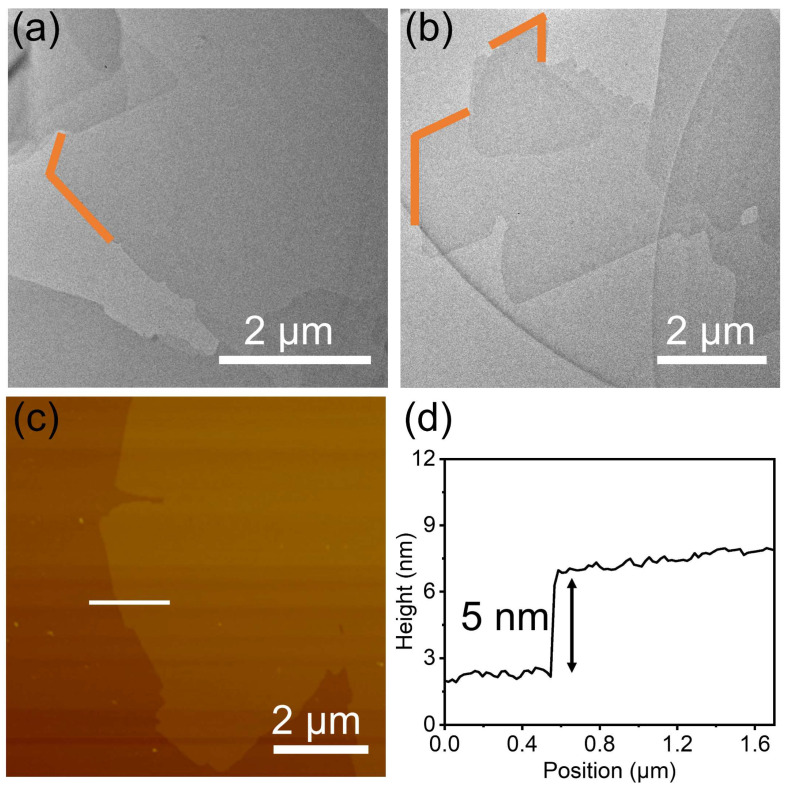
(**a**,**b**) are TEM images of **P1**. Orange segments mark angles. (**c**) AFM image of **P1** on SiO_2_/Si. The white line marks the position of the cross-section. (**d**) Cross-sectional analysis along the white line in (**c**).

The TEM images show that the film was ultrathin and continuous, with a sheet-like morphology. Several angles are denoted by orange segments in Figure 2a,b, which reveal the rigidity of the 2D polymer film **P1** to some extent. The image of **P1** film can also be obtained on a copper TEM grid (Figure S3). On the whole, the 2D polymer film **P1** displayed sharply defined, regular edges, and very few wrinkles, which suggests high crystallinity. Notably, the ultrathin film does not burn immediately under the focused beams during TEM, which indicates the good stability of the films. The AFM image indicates that the 2D polymer film **P1** has a rather flat and uniform surface (Figure 2c). The carbon–carbon-linked ultrathin 2D polymer film **P1** possesses a large-area (over 50 μm^2^ in domain size), and the average thickness of the film is approximately 5 nm (Figure 2d).

**Figure 3:**
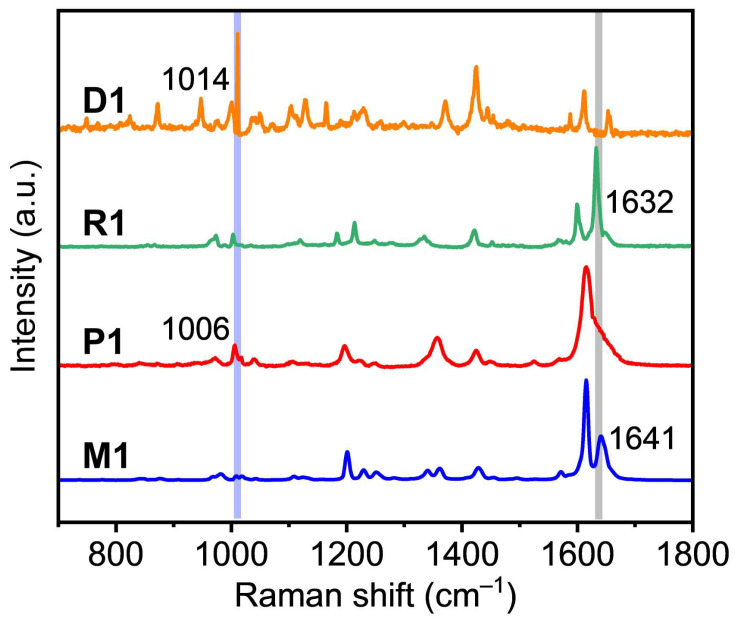
Raman spectra of **D1**, **R1**, **P1,** and **M1** on SiO_2_/Si wafer. The blue and gray vertical lines correspond to the characteristic peak positions of cyclobutene and -C=C- bonds, respectively.

The Raman spectra further revealed changes in the characteristic functional group peaks before and after [2 + 2] cycloaddition at the interface. As we know, Raman is a powerful tool for characterizing chemical information with high sensitivity, spatial resolution, and chemical selectivity. To prove the successful photocycloaddition, the model compound (**R1**) and the corresponding dimer (**D1**) were measured by Raman spectroscopy. As shown in Figure 3, the Raman spectra of **R1** and **D1** have obvious changes before and after dimerization. Similar changes are also observed in the Raman spectra of **M1** and **P1**. The peak at 1632 cm^−1^ of **R1** and the peak at 1641 cm^−1^ of **M1** correspond to the stretching vibrations of single -C=C- bonds, which disappeared in the prepared **D1** and **P1**. Meanwhile, new peaks were observed at 1006 cm^−1^ for **P1** and at 1014 cm^−1^ for **D1**, which mainly belong to the stretching vibrations of the cyclobutene. In conclusion, the Raman spectra confirmed that the polymerization was successful. Moreover, also **P1** could be depolymerized back to **M1** under heating conditions, which was measured by Raman spectra (Figure S4). With the extension of the heating time, the intensity of the -C=C- bonds peak became stronger, whereas the intensity of the cyclobutene peak became weaker gradually, implying the stepwise elimination of cyclobutane rings and resulting in the depolymerization process. To obtain a further understanding of the bonding properties of **P1**, the chemical bonding analysis was performed using the XPS spectra (Figure S5). According to the XPS spectra, the results of the multilayers indicate unambiguously that the **P1** film is mainly composed of carbon, nitrogen, fluorine, oxygen, and silicon (Figure S5a). As the substrate is SiO_2_/Si, silicon was observed. Further analysis from the high-resolution C 1s spectra shows that C=C, C-F, C-N, and C-C make the major contributions to the Gaussian curves (Figure S5b). Additionally, C-O and C=O forms are detected. The C 1s peak can be mainly deconvoluted into three subpeaks at 284.2, 284.7, 285.4, 286.2, 287.3, and 288.8 eV, which have been assigned to C 1s orbitals of C-N (sp^3^), C=C (sp^2^), C-F (sp^2^), C-C (sp^3^), C-O, and C=O, respectively. The area ratio of C-N/C=C/C-F/C-C is around 3:9:4:2, which is in agreement with the chemical composition of **P1**.

**Figure 4:**
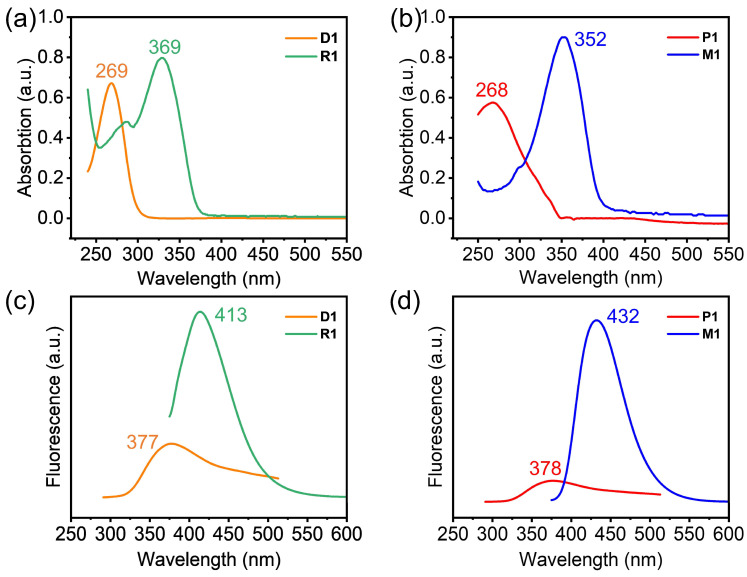
(**a**,**b**) are the UV-vis absorption spectra and (**c**,**d**) fluorescence spectra of **D1**, **R1**, **P1**, and **M1**.

Besides the evidence from the Raman spectra and XPS spectra, the photophysical property further confirms the success of the photo-polymerization. The UV-vis absorption and fluorescence spectrum changed significantly after photo-polymerization. The UV-vis absorption spectra of **D1** and **R1** display the maximum absorption peaks located at around 269 and 369 nm, respectively (Figure 4a). **P1** and **M1** displayed similar UV absorption peaks at around 268 nm and 352 nm (Figure 4b). Both **D1** and **P1** have a significant blue shift, which is due to the interruption of conjugation via the successful photochemically triggered [2 + 2] cycloaddition. In the fluorescence spectrum, **D1** and **R1** showed emission peaks at around 377 nm and 413 nm (Figure 4c), while the fluorescence intensity of **D1** became significantly weakened. The fluorescence emission peaks of **P1** and **M1** were located at 378 nm and 432 nm (Figure 4d). A similar fluorescence change phenomenon was also observed for **M1** and **P1**. The fluorescence intensity of **P1** decreased much more markedly compared with that of **M1**. After photochemically triggered [2 + 2] cycloaddition, the emission peaks of **P1** and **D1** also showed an obvious blue shift.

## 3. Materials and Methods

### 3.1. Materials

Unless otherwise specified, the starting materials were purchased commercially and used as received without further purification. Iodobenzene and 4′-Iodoacetophenone were supplied by Aladdin (Shanghai, China). We purchased 2,3,4,5,6-pentafluorostyrene, *N*,*N*-dimethylformamide (DMF), dichloromethane (DCM), hexane, absolute ethanol, ethyl acetate, thionyl chloride, KOH, K_2_CO_3_, Na_2_SO_4_, Pd(OAc)_2_, and 2-(2-methoxyethoxy)-*N*,*N*-bis [2-(2-methoxyethoxy)ethyl]ethanamine from Energy Chemical (Shanghai, China). Nitrogen stored in a high-pressure gas cylinder was ordered from Dehai Gas (Qingdao, China). All of the aqueous solutions were prepared with Milli-Q water.

### 3.2. Characterizations

Using silica gel of 200–300 mesh, column chromatography was performed. The TLC analysis was conducted on precoated silica gel plates (0.2 mm in thickness). The ^1^H, ^13^C, and ^19^F NMR spectra were collected in the indicated solvents at room temperature using a 400 MHz spectrometer (Bruker AVANCE NEO 400 Ascend, Bruker, Bremen, Germany). Matrix-Assisted Laser Desorption Ionization—Time of Flight (MALDI-TOF) mass spectrometry analysis was performed on a Bruker Microflex-LRF mass spectrometer (Bruker, Bremen, Germany) in positive ion reflector mode using dichloromethane as the solvent. Using a UV-Vis spectrometer (HITACHI, 3900, Tokyo, Japan), the absorbance of the liquid UV-VIS was measured. An F-2700 fluorescence spectrometer (HITACHI) was used to record the fluorescence spectra. A 20 kV, 30 mA X-ray diffractometer (XRD, X-Pert, Panalytical, Almelo, The Netherlands) with Mo Kα radiation (40 kV, 30 mA) was used to determine the crystal structure. The transmission electron microscope used in this study was a JEM-2100 electron microscope (JEOL Japan Electronics Co., Ltd., Tokyo, Japan) with a 200 kV accelerating voltage. The images were obtained using the Nanoscope V controller attached to Bruker Multimode 8 Atomic Force Microscope (AFM) (Bruker, Bremen, Germany). Samples were imaged by operating it in the PF-QNM mode at a scan rate of 1.0 Hz. Raman spectra were investigated using a HORIBA Raman spectrometer (LabRAM HR Evolution, Paris, France) with an excitation wavelength at 633 nm and a spot diameter of 1 μm. The LB trough used in the experiment was KSV Mediumtrough (KSV NIMA, Goteborg, Sweden). Two symmetrical barriers were made of Delrin, and the trough was made of Teflon. For BAM measurements, a 659 nm laser from the MicroBAM (KSV NIMA, Goteborg, Sweden) was used. The Langmuir trough (Teflon) was fitted with black glass to permit total reflection without films. Optical microscopes (Vision Engineering Co., Goteborg, Sweden) were used to acquire images, which were then captured using CCD cameras. X-Ray photoelectron spectroscopy was performed on a Thermo Scientific ESCALab 250Xi instrument (Waltham, MA, USA). The parameters were as follows: Al Kα (1486.6 eV, 150 W) radiation was used as the X-ray source, the vacuum degree of the chamber was 3.6 × 10^−9^ mbar, and the scan range was −10~1350 eV.

### 3.3. Synthesis Details

We synthesized 1,3,5-tris(4-iodophenyl)benzene (**2**) according to the previously reported procedures [66]. We prepared 2,3,5,6-tetrafluoro-*N*,*N*-dimethyl-4-vinylaniline (**1**), and the model compound (**R1**), dimer (**D1**), and monomer (**M1**) according to previously described methods [50,67]. The preparation of the 2D films using the Langmuir–Blodgett (LB) trough was conducted according to a previously reported study [68].

#### 3.3.1. Compound (**1**)

We mixed 2,3,4,5,6-pentafluorostyrene (1.94 g, 10 mmol), KOH (1.40 g, 25 mmol), and *N*,*N*-dimethylformamide (5.5 mL) in a 25 mL pear flask equipped with one magnetic stir bar under an atmosphere of air, and then heated the solution to 120 °C for 24 h. After cooling the mixture to room temperature, 20 mL of water was added to the mixture under stirring, and then extracted with ethyl acetate (2 × 50 mL). The combined organic layers were dried with anhydrous sodium sulfate Na_2_SO_4_. The vacuum was used to remove the solvent. Additionally, the crude product was purified by column chromatography, eluting with a 10:1 mixture of hexane and CH_2_Cl_2_, to give compound (**1**) (2.0 g, 95%) as a sticky colorless oil. ^1^H-NMR (400 MHz, CDCl_3_): δ 2.96 (t, *J* = 2.1 Hz, 6H), 5.55 (d, *J* = 11.9 Hz, 1H), 5.97 (d, *J* = 17.9 Hz, 1H), 6.62 (dd, *J* = 18.0, 11.9 Hz, 1H).

#### 3.3.2. Compound **R1**

A 50 mL pear flask equipped with a magnetic stir bar was charged with Iodobenzene (4.08g, 20 mmol), compound (**1**) (4.38 g, 20.0 mmol), anhydrous DMF (10 mL), K_2_CO_3_ (3.50 g, 25.0 mmol), and 2-(2-methoxyethoxy)-*N*,*N*-bis[2-(2-methoxyethoxy)ethyl]ethanamine (0.16 g, 0.50 mmol). After being degassed thoroughly with nitrogen, Pd(OAc)_2_ (22 mg, 0.1 mmol) was added. The reaction was gradually warmed to 110 °C in a nitrogen atmosphere for 24 h. We cooled the mixture to room temperature, water (100 mL) was added slowly, and then the mixture was extracted with CH_2_Cl_2_ (2 × 200 mL). Over anhydrous Na_2_SO_4_, the organic layers were dried. The vacuum was used to remove the solvent. The crude product was purified by flash column chromatography, eluting with a 3:1 mixture of hexane and CH_2_Cl_2_ to give **R1** as a light yellow solid (4.70 g, 80% yield). ^1^H-NMR (400 MHz, CDCl_3_): δ 2.99 (t, *J* = 2.1 Hz, 6H), 7.01 (d, *J* = 16.8 Hz, 1H), 7.30 (d, *J* = 7.3 Hz, 1H), 7.35 (d, *J* = 3.8 Hz, 1H), 7.38 (d, *J* = 5.1 Hz, 2H), δ 7.52 (d, *J* = 7.6 Hz, 2H). ^13^C-NMR (400 MHz, CDCl_3_): δ 145.5 (t), 143.1 (t), 142.5 (t), 139.8 (t), 136.1 (s), 133.5 (t), 128.7 (t), 127.7 (s), 127.2 (s), 125.5 (s) 113.2 (s), 108.1 (t), 42.2 (s). ^19^F-NMR (376 MHz, CDCl_3_): δ −152.82 (m, 2F), −145.24 (m, 2F).

#### 3.3.3. Compound **D1**

A saturated dichloromethane solution of **R1** in a 3 mL flask was irradiated by 450 nm for 2 h. Then, the solution was concentrated under reduced pressure. **D1** was obtained after being washed with petroleum ether (80% yield). ^1^H-NMR (400 MHz, CDCl_3_): δ 7.28–7.13 (m, 10H), 4.95–4.89 (m, 2H), 4.84–4.77 (m, 2H), 2.86 (t, *J* = 2.0 Hz, 12H). ^13^C-NMR (400 MHz, CDCl_3_): δ 146.8 (s), 144.4 (s), 143.2 (s), 140.7 (s), 140.1 (s), 129.5 (s), 128.2 (s), 127.0 (s), 126.5 (s), 110.7 (t), 45.5 (d), 43.2 (t), 38.79 (s). ^19^F-NMR (376 MHz, CDCl_3_): δ −143.96 (m, 4F), −152.35 (m, 4F). MS (MALDI-TOF): calcd. for C_32_H_26_F_8_N_2_: 590.56, found 591.22 [M+H]^+^.

#### 3.3.4. Compound **R2**

A three-necked flask equipped with a temperature prober was charged with absolute ethanol (4.4 mL) and 4’-iodoacetophenone (3.7 g, 15.0 mmol). During vigorous stirring, thionyl chloride (1.8 mL, 25.0 mmol) was added in a dropwise way, and then the reaction mixture was refluxed for 2h. After the completion of the reaction, the reaction mixture was neutralized with saturated sodium carbonate. The precipitate was collected by filtration, washed with water and ethanol, and dried in a vacuum. The crude product was purified by flash column chromatography with petroleum ether to give **R2** as an earthy yellow solid. (2.20 g, 64%).^1^H-NMR (400 MHz, CDCl_3_): δ 7.70 (d, *J* = 8.5 Hz, 6H), 7.86 (d, *J* = 8.4 Hz, 6H), 7.90 (s, 3H)

#### 3.3.5. Compound **M1**

A 50 mL pear flask equipped with a magnetic stir bar was charged with compound (**2**) (4.51 g, 6.6 mmol), compound **R1** (4.50 g, 20.0 mmol), anhydrous DMF (10 mL), K_2_CO_3_ (3.50 g, 12.5 mmol), and 2-(2-methoxyethoxy)-*N*,*N*-bis[2-(2- methoxyethoxy)ethyl]ethanamine (0.16 g, 0.50 mmol). After being degassed thoroughly with nitrogen, Pd(OAc)_2_ (22 mg, 0.1 mmol) was added. The reaction was gradually heated up to 110 °C for 24 h in a nitrogen atmosphere. We cooled the mixture to room temperature, water (100 mL) was added slowly, and then the mixture was extracted with CH_2_Cl_2_ (2 × 200 mL). Over anhydrous Na_2_SO_4_, the organic layers were dried. The vacuum was used to remove the solvent. The crude product was purified by flash column chromatography, eluting with a 5:1 mixture of hexane and CH_2_Cl_2_ to give **M1** as a light yellow solid (3.73 g, 60% yield). ^1^H-NMR (400 MHz, CDCl_3_): δ 7.83 (s, 3H), 7.73 (d, *J* = 8.4 Hz, 6H), 7.65 (d, *J* = 8.4 Hz, 6H), 7.43 (d, *J* = 16.7 Hz, 3H), 7.09 (d, *J* = 16.7 Hz, 3H), 3.00 (t, *J* = 2.0 Hz, 18H). ^13^C-NMR (400 MHz, CDCl_3_): δ 146.6 (s), 144.2 (s), 143.3 (s), 141.8 (s), 141.0 (s), 140.7(s), 136.6 (s), 133.8 (t), 129.8 (s), 127.6 (s), 127.2(s), 124.8 (s), 114.4 (s), 108.9 (s), 43.3 (s). ^19^F-NMR (376 MHz, CDCl_3_): δ −152.85 (s, 6F), −145.03 (s, 6F). MS (MALDI-TOF): calcd. for C_54_H_39_F_12_N_3_: 957.91, found 958.11 [M].

#### 3.3.6. The Preparation of the 2D Polymer Film **P1**

Deionized water was uniformly injected into the prepared test bench samples. Using a micro-liter syringe, 250 μL of a 1.0 × 10^−5^ M solution of **M1** in CH_2_Cl_2_ was applied to the water surface. Following the evaporation of the solvent for 20 min, compression was conducted at 3 mm min^−1^ until a surface pressure of 41 mN m^−1^ was reached. Photochemically triggered polymerization then took place using a home-built 450 nm LED lamp (power: 36 W) for 2 h (LED lamp was homemade by ourselves, Qingdao, China). A constant rate of 0.5 mm per minute was used to lift up the pre-submerged substrate (e.g., silicon wafer). As soon as the drying process has been completed, the collected **P1** film was subjected to TEM, Raman, XPS, and AFM for characterization.

#### 3.3.7. Film **P1** Transfer and Spanning

The transfer pressure on the substrates was set to 41 mN m^−1^. TEM grids made up of copper with a mesh size of 300 (XXBR, T10023, China) were placed on the surface. A clean white paper was then gently placed on the grids until the soaked paper had TEM grids attached to it. The paper along with the grids on it was then removed and left at room temperature until it was dry. For the vertical transferring of the films, the substrate was initially immersed horizontally in the water subphase. After 2 h of polymerization at 41 mN m^−1^, the substrate was pulled up at the constant rate of 0.5 mm min^−1^. The substrate with the **P1** films was put into a sample bottle with CH_2_Cl_2_ and sonicated for 10 min. The vast majority of the **P1** films were dispersed into the solvent by ultrasonication. We repeated similar operations several times.

## 4. Conclusions

In summary, a carbon–carbon-linked, ultrathin 2D polymer film was prepared in a well-controlled manner at the air/water interface. The self-assembly process of the monomers was caused by D-A and π-π interactions, which played a vital role in the photo-polymerization. Representative images obtained by BAM, AFM, and TEM revealed that the ultrathin film was uniform, continuous, and had a large area. Raman spectrum and XPS spectroscopy provided direct evidence of the successful polymerization of the 2D polymer film via photochemically triggered [2 + 2] cycloaddition. This work not only provides a simple and powerful approach for constructing carbon–carbon-linked 2D polymer films, but it also reveals a guide for the molecular design strategy of 2D polymer films.

## Data Availability

Not applicable.

## Supplementary Materials

The following supporting information can be downloaded at: https://www.mdpi.com/article/10.3390/molecules28041930/s1, Scheme S1: Synthesis of molecules **R1** and **M1**; Figure S1: Surface pressure-mean molecular area (SP-MMA) isotherm of compressing **M1** at a compression rate of 3 mm/min; Figure S2: BAM images of the process of compressing **M1** in the SP-MMA isotherm study; Figure S3. Free-standing **P1** film on a copper TEM grid. Figure S4: Raman spectrum of the depolymerization process of **P1**. Figure S5: (a) XPS survey spectra of **P1** and (b) XPS C 1s spectra of **P1** on SiO_2_/Si wafer. Figure S6: Mass spectrum of the compound **D1**; Figure S7: Mass spectrum of the compound **M1**; Figure S8:^1^H NMR spectrum of compound **(1**); Figure S9: ^1^H NMR spectrum of compound **(2**); Figure S10: ^1^H NMR spectrum of the compound **R1**; Figure S11: ^13^C NMR spectrum of the compound **R1**; Figure S12: ^19^F NMR spectrum of the compound **R1**; Figure S13: ^1^H NMR spectrum of the compound **D1**; Figure S14: ^13^C NMR spectrum of the compound **D1**; Figure S15: ^19^F NMR spectrum of the compound **R1**; Figure S16: ^1^H NMR spectrum of the compound **M1**; Figure S17: ^13^C NMR spectrum of the compound **M1**; Figure S18: ^19^F NMR spectrum of the compound **M1**; Table S1: The single crystal date of **R1**.

## Sample Availability

Samples of the compounds are available from the authors.
