# Supplementary material for: Interfacial Synthesis of an Ultrathin Two-Dimensional Polymer Film via [2 + 2] Photocycloaddition
Source: Molecules. 2023 Feb 17;28(4):1930. doi: 10.3390/molecules28041930 (PMC9965025; doi:10.3390/molecules28041930)
Supplement: Supplementary file 1 [file molecules-28-01930-s001.zip › molecules-2183358-supplementary.pdf]

Supporting Information

Interfacial Synthesis of an Ultrathin Two-Dimensional Polymer Film via [2 + 2] Photocycloaddition

Yanqi Ban [1], Hui Wang [2], Zixuan Xiao [1], Lishui Sun [1], Qingyan Pan [1,*] and Yingjie Zhao [1,*]

[1] Engineering Research Center of High Performance Polymer and Molding Technology, Ministry of Education, College of Polymer Science and Engineering, Qingdao University of Science and Technology, Qingdao 266042, China
[2] College of Chemical Engineering, Qingdao University of Science and Technology, Qingdao 266042, China
* Correspondence: qingyanpan@qust.edu.cn (Q.P.); yz@qust.edu.cn (Y.Z.)

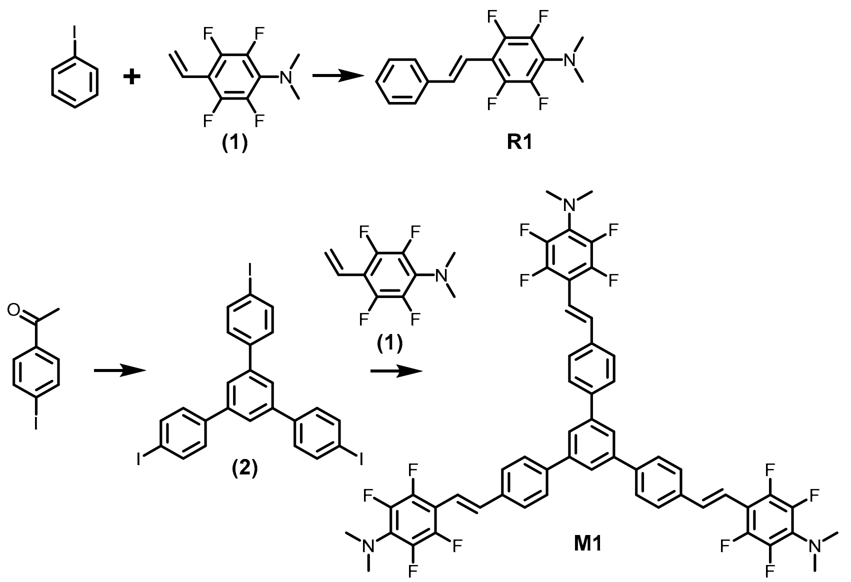

Scheme S1. Synthesis of molecules **R1** and **M1.**

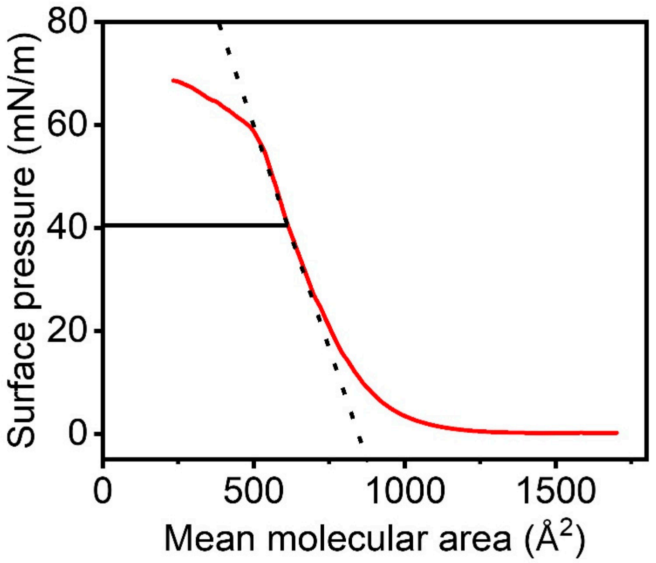

Figure S1. Surface pressure-mean molecular area (SP-MMA) isotherm of compressing **M1** with a compression rate of 3 mm/min.

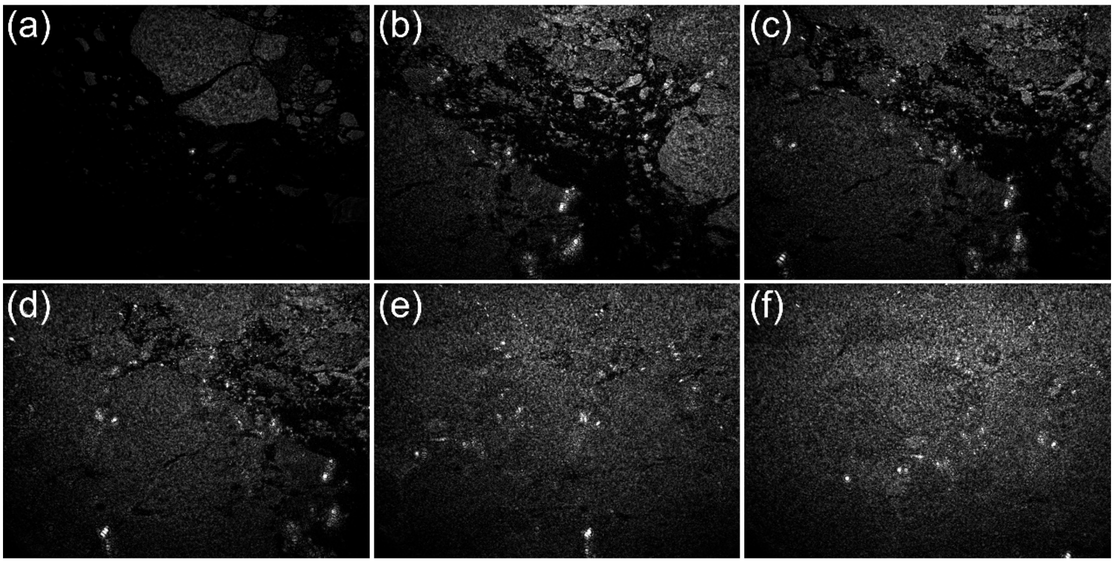

Figure S2. BAM images of the process of compressing **M1** in the SP-MMA isotherm study.

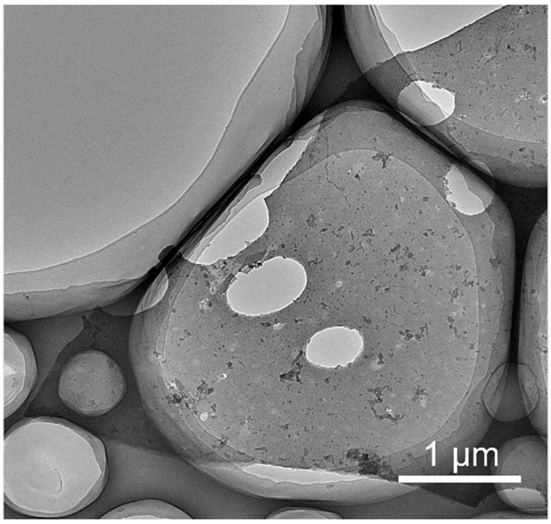

Figure S3. Free-standing **P1** film on a copper TEM grid.

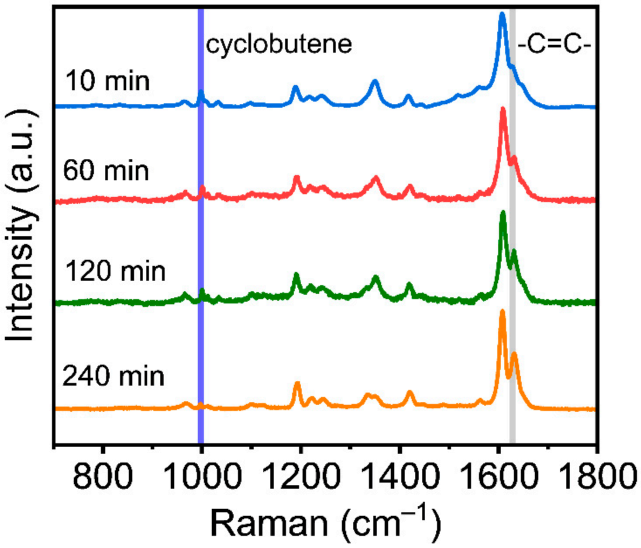

Figure S4. Raman spectrum of the depolymerization process of **P1**.

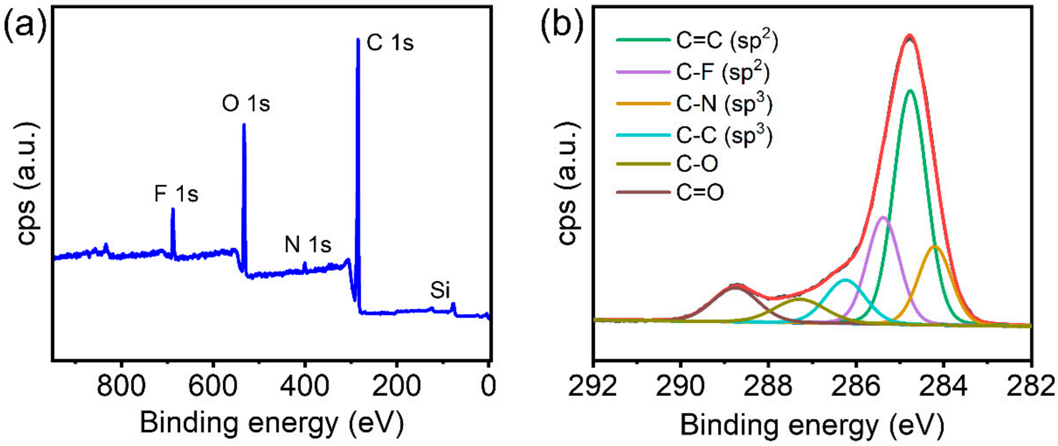

Figure S5. (a) XPS survey spectra of **P1** and (b) XPS C 1s spectra of **P1** on SiO_2/Si wafer.

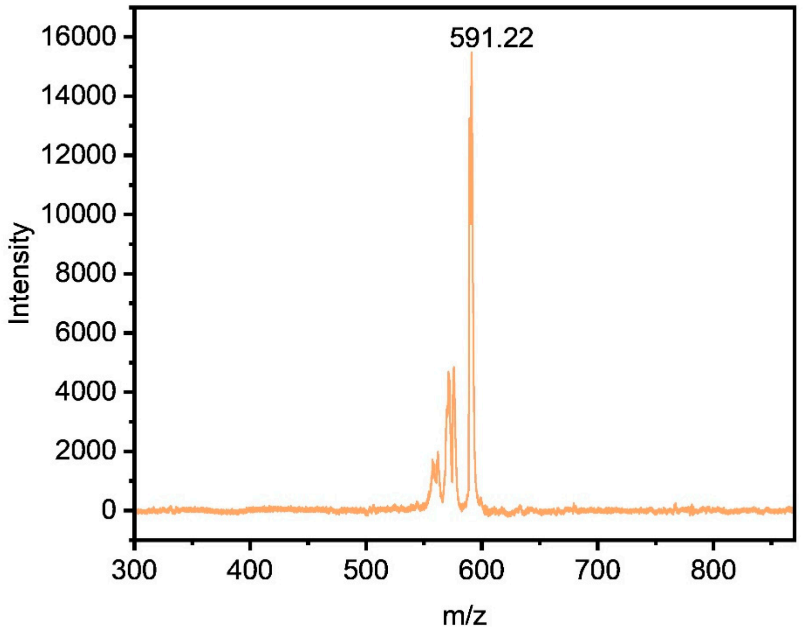

Figure S6. Mass spectrum of the compound **D1**.

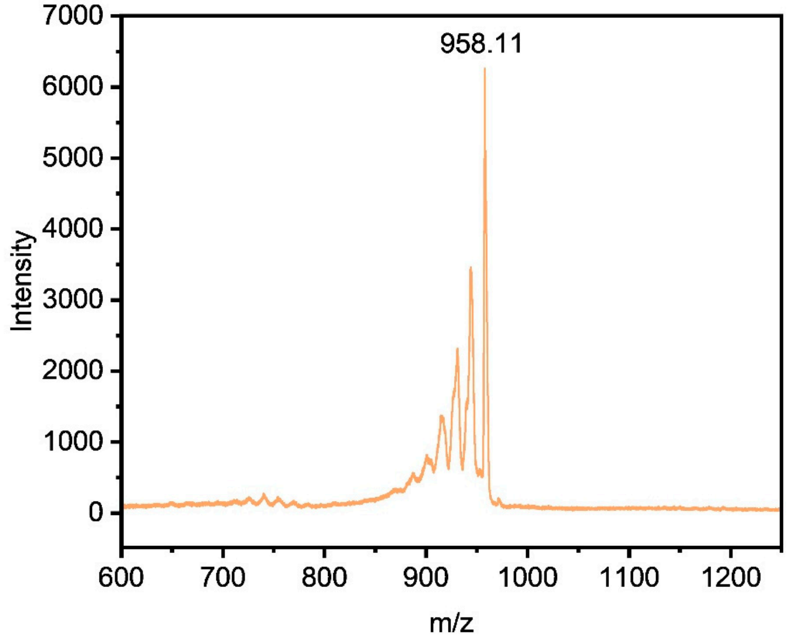

Figure S7. Mass spectrum of the compound **M1**.

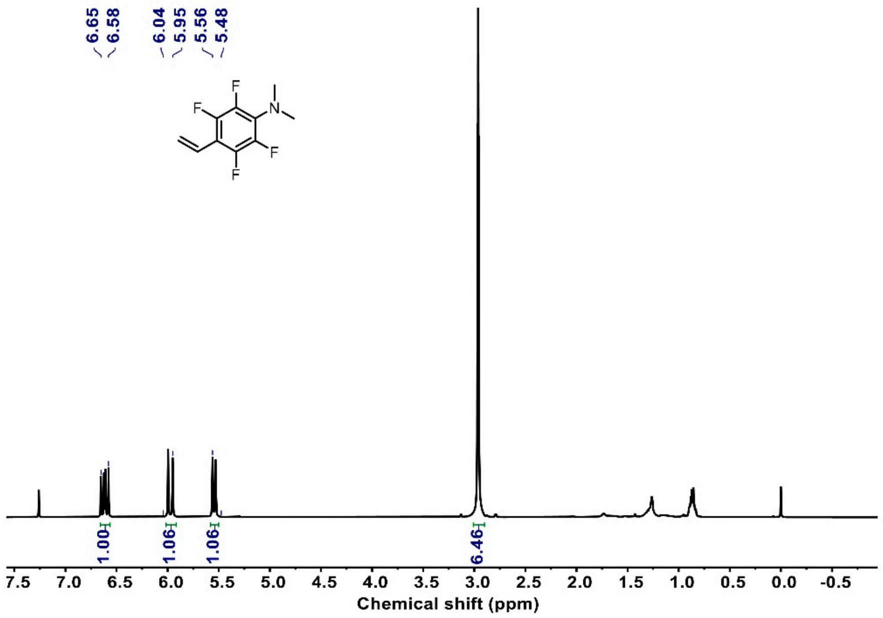

Figure S8. ^{1}H NMR spectrum of compound (**1**).

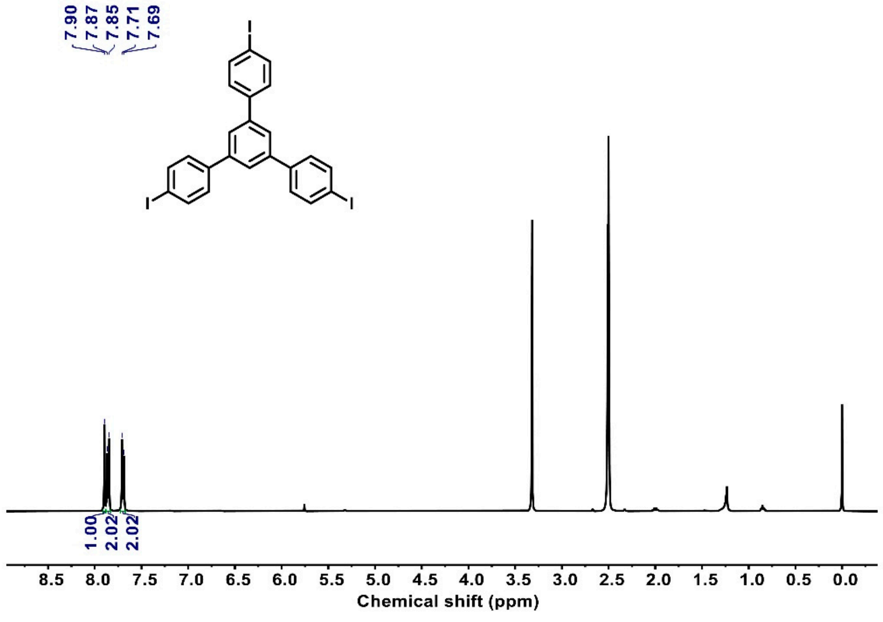

Figure S9. ^{1}H NMR spectrum of compound (**2**).

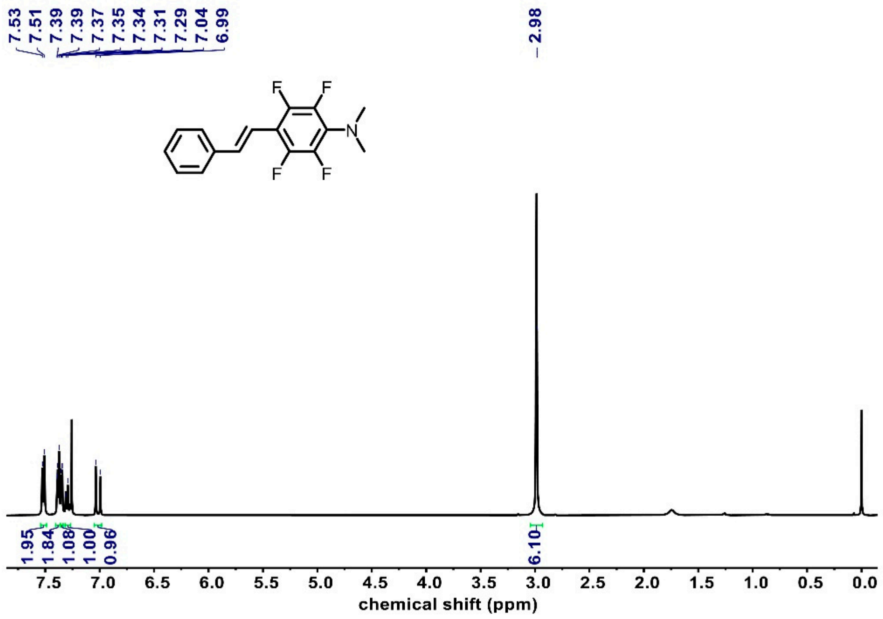

Figure S10. ^{1}H NMR spectrum of the compound **R1**.

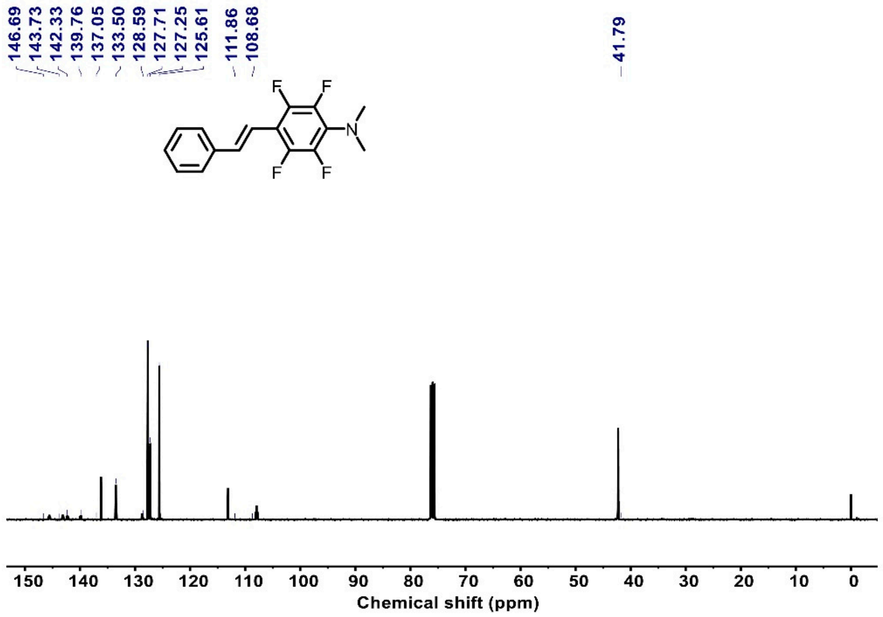

Figure. S11. ^{13}C NMR spectrum of the compound **R1**.

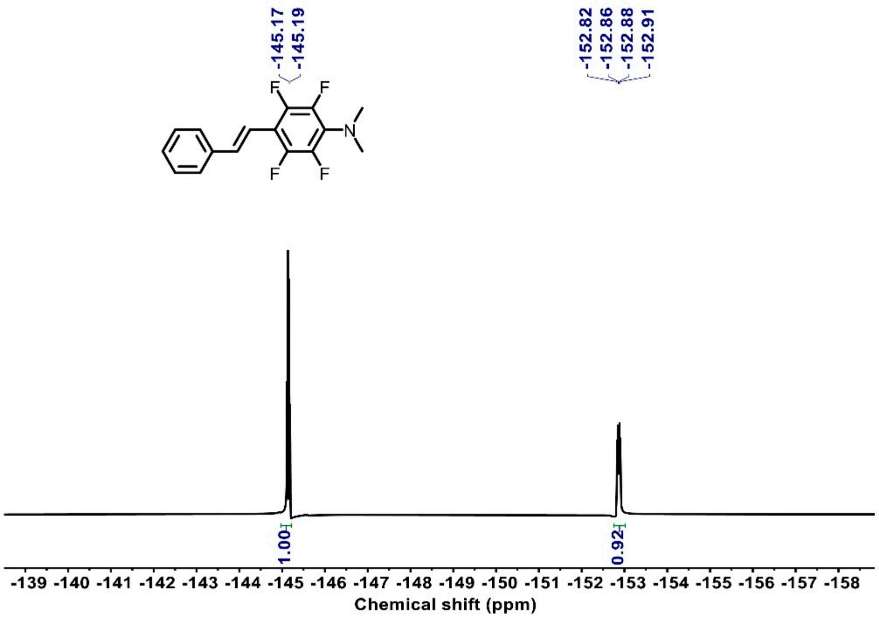

Figure S12. ^{19}F NMR spectrum of the compound **R1**.

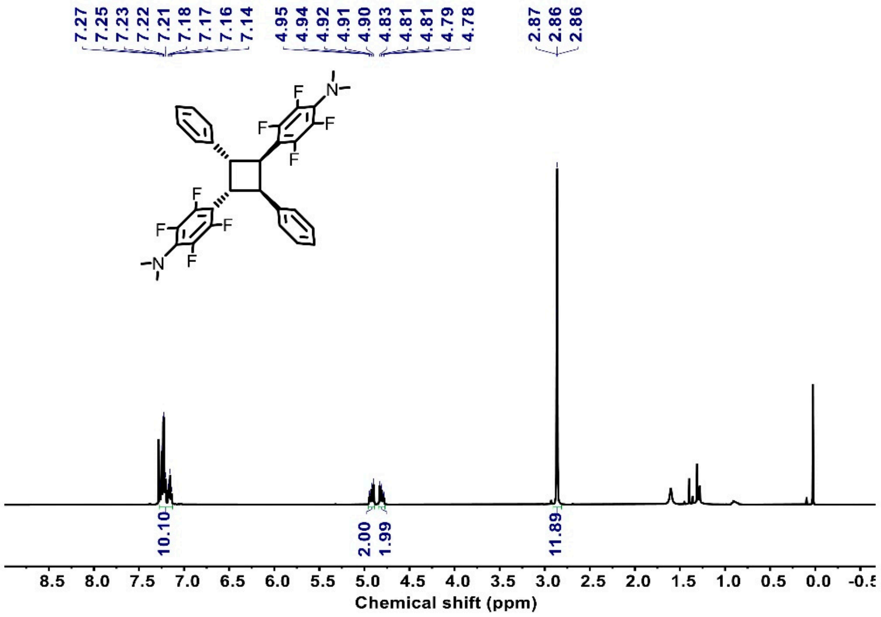

Figure S13. ^{1}H NMR spectrum of the compound **D1**.

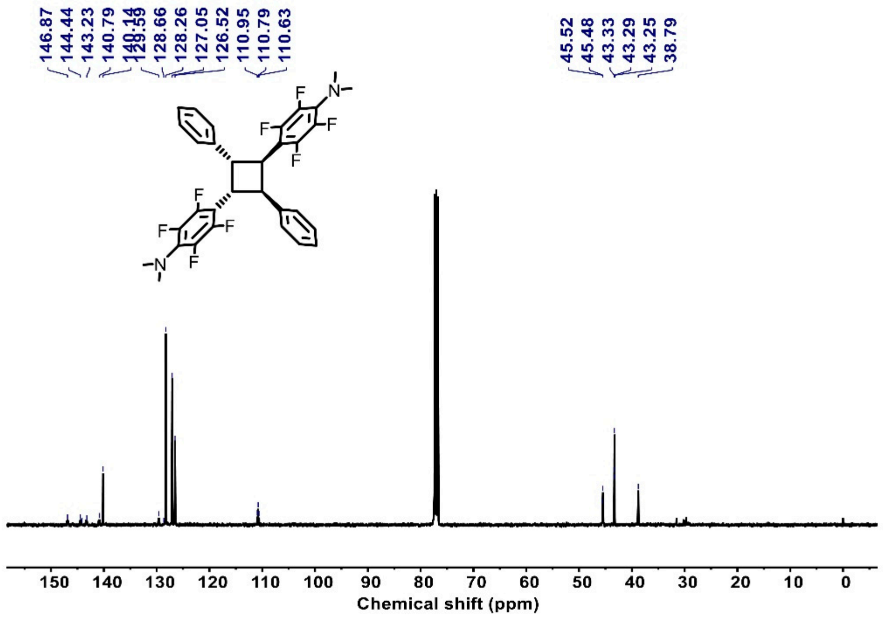

Figure S14. ^{13}C NMR spectrum of the compound **D1**.

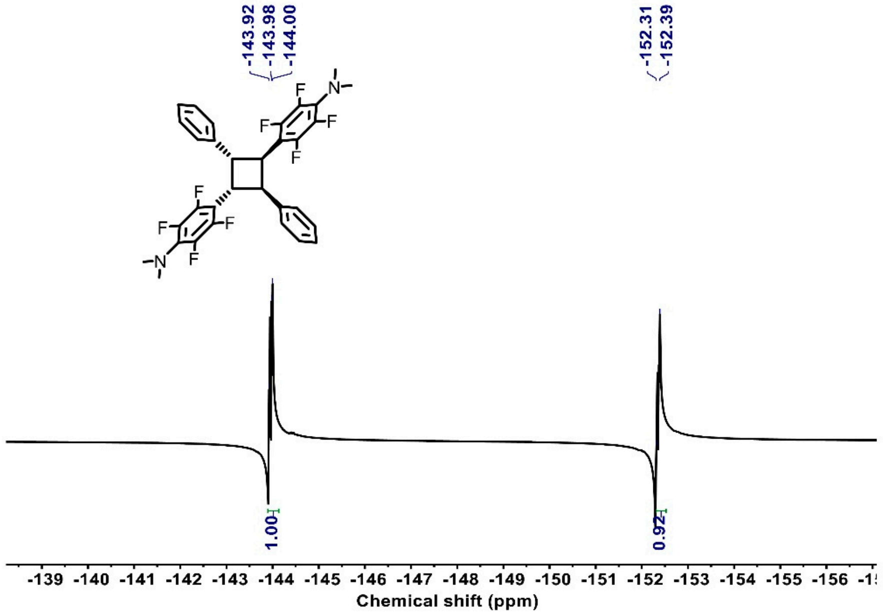

Figure S15. ^{19}F NMR spectrum of the compound **R1**.

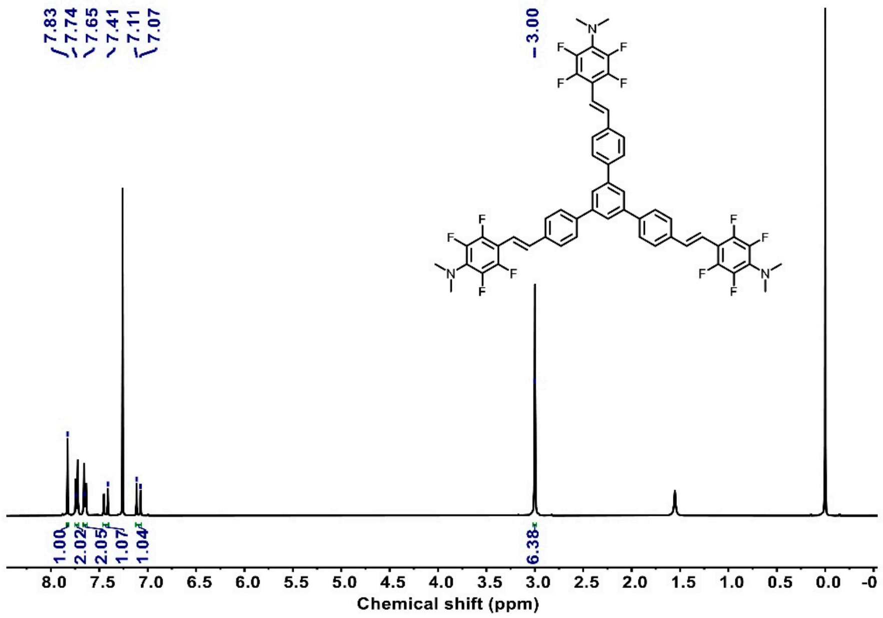

Figure S16. ¹H NMR spectrum of the compound **M1**.

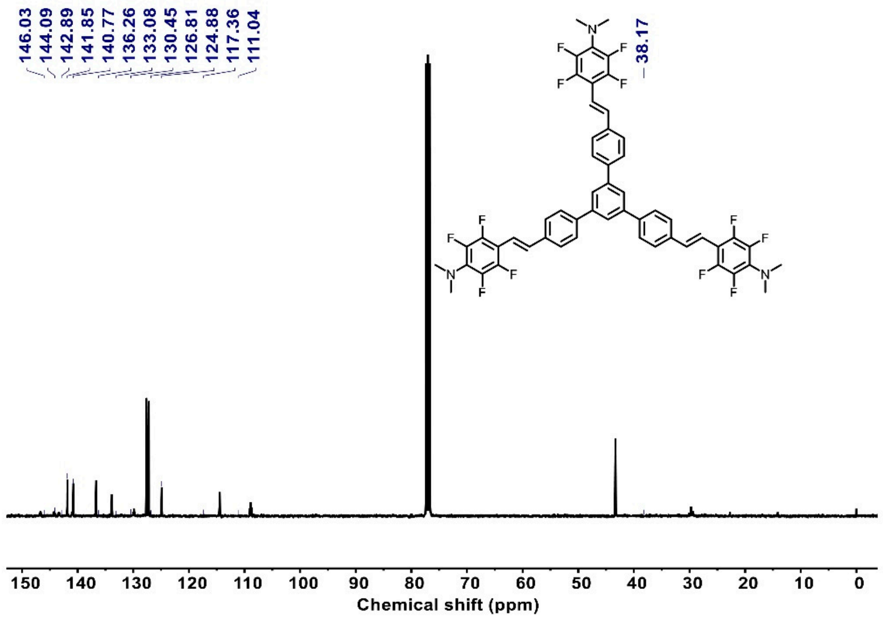

Figure S17. ¹³C NMR spectrum of the compound **M1**.

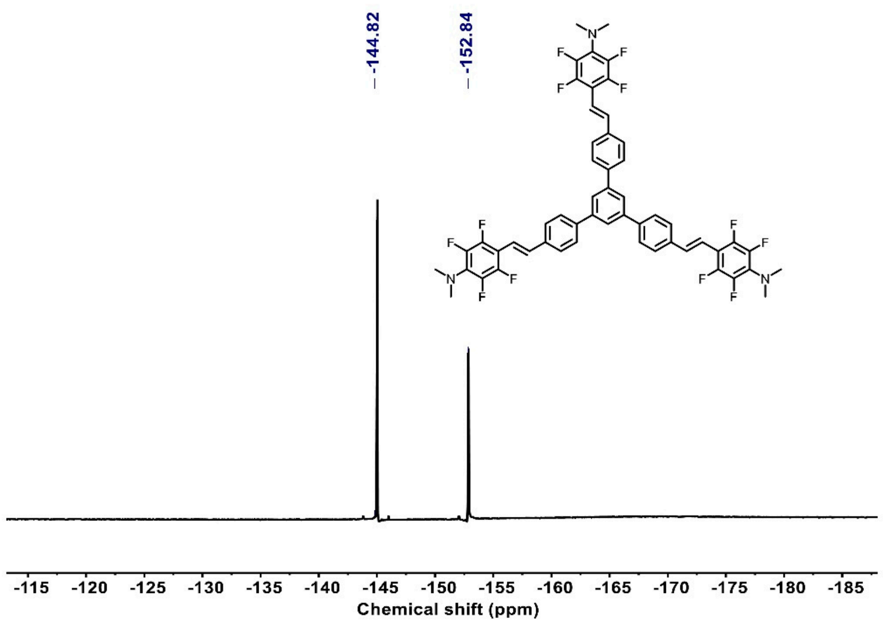

Figure S18. ^{19}F NMR spectrum of the compound **M1**.

Table S1: The single crystal date of **R1.**

Identification code	MX8069
CCDC number	2226830
Empirical formula	C16H13F4N
Formula weight	295.27
Temperature/K	170.00(10)
Crystal system	monoclinic
Space group	P21/c
a/Å	10.4213(4)
b/Å	7.5800(3)
c/Å	17.4234(7)
α/°	90
β/°	101.852(4)
γ/°	90
Volume/Å3	1346.99(9)
Z	4
ρcalcg/cm3	1.456
μ/mm-1	0.124
F(000)	608.0
Crystal size/mm3	0.58 × 0.43 × 0.35
Radiation	Mo Kα (λ = 0.71073)
2Θ range for data collection/	5.562 to 60.534
Index ranges	-14 ≤ h ≤ 9, -7 ≤ k ≤ 10, -23 ≤ l ≤ 22
Reflections collected	10583
Independent reflections	3592 [Rint = 0.0141, Rsigma = 0.0154]
Data/restraints/parameters	3592/0/211
Goodness-of-fit on F2	1.079
Final R indexes [I>=2σ (I)]	R1 = 0.0682, wR2 = 0.1801
Final R indexes [all data]	R1 = 0.0814, wR2 = 0.1895
Largest diff. peak/hole / e Å-3	0.52/-0.82
